# Molecular Docking and Dynamics Simulation Studies Predict Munc18b as a Target of Mycolactone: A Plausible Mechanism for Granule Exocytosis Impairment in Buruli Ulcer Pathogenesis

**DOI:** 10.3390/toxins11030181

**Published:** 2019-03-25

**Authors:** Samuel K. Kwofie, Bismark Dankwa, Kweku S. Enninful, Courage Adobor, Emmanuel Broni, Alfred Ntiamoah, Michael D. Wilson

**Affiliations:** 1 Department of Biomedical Engineering, School of Engineering Sciences, College of Basic and Applied Sciences, University of Ghana, P.O. Box LG77, Legon, Accra, Ghana; SKKwofie@ug.edu.gh (S.K.K.); antiamoah890@gmail.com (A.N.); 2 West African Center for Cell Biology of Infectious Pathogens, Department of Biochemistry, Cell and Molecular Biology, College of Basic and Applied Sciences, University of Ghana, P.O. Box LG 25, Legon, Accra, Ghana; 3 Department of Medicine, Loyola University Medical Center, Maywood, IL 60153, USA; 4 Department of Parasitology, Noguchi Memorial Institute for Medical Research, University of Ghana, P.O. Box LG 581, Legon, Accra, Ghana; dankwabismark52@gmail.com (B.D.); kweku765@gmail.com (K.S.E.); adoborcourage@gmail.com (C.A.); ebroni002@st.ug.edu.gh (E.B.)

**Keywords:** Buruli ulcer, mycolactone, chaperone proteins, SNARE proteins, Munc18b, Sec61, AT2R, WASP, molecular docking, molecular dynamics

## Abstract

Ulcers due to infections with *Mycobacterium ulcerans* are characterized by complete lack of wound healing processes, painless, an underlying bed of host dead cells and undermined edges due to necrosis. Mycolactone, a macrolide produced by the mycobacterium, is believed to be the toxin responsible. Of interest and relevance is the knowledge that Buruli ulcer (BU) patients remember experiencing trauma previously at the site of the ulcers, suggesting an impairment of wound healing processes, the plausible effect due to the toxin. Wound healing processes involve activation of the blood platelets to release the contents of the dense granules mainly serotonin, calcium ions, and ADP/ATP by exocytosis into the bloodstream. The serotonin release results in attracting more platelets and mast cells to the wound site, with the mast cells also undergoing degranulation, releasing compounds into the bloodstream by exocytosis. Recent work has identified interference in the co-translational translocation of many secreted proteins via the endoplasmic reticulum and cell death involving Wiskott-Aldrich syndrome protein (WASP), Sec61, and angiotensin II receptors (AT2R). We hypothesized that mycolactone by being lipophilic, passively crosses cell membranes and binds to key proteins that are involved in exocytosis by platelets and mast cells, thus inhibiting the initiation of wound healing processes. Based on this, molecular docking studies were performed with mycolactone against key soluble n-ethylmaleimide-sensitive factor attachment protein receptor (SNARE) proteins and regulators, namely Vesicle-associated membrane protein (VAMP8), Synaptosomal-associated protein (SNAP23, syntaxin 11, Munc13-4 (its isoform Munc13-1 was used), and Munc18b; and also against known mycolactone targets (Sec61, AT2R, and WASP). Munc18b was shown to be a plausible mycolactone target after the molecular docking studies with binding affinity of −8.5 kcal/mol. Structural studies and molecular mechanics Poisson-Boltzmann surface area (MM-PBSA) binding energy calculations of the mycolactone and Munc18b complex was done with 100 ns molecular dynamics simulations using GROMACS. Mycolactone binds strongly to Munc18b with an average binding energy of −247.571 ± 37.471 kJ/mol, and its presence elicits changes in the structural conformation of the protein. Analysis of the binding interactions also shows that mycolactone interacts with Arg405, which is an important residue of Munc18b, whose mutation could result in impaired granule exocytosis. These findings consolidate the possibility that Munc18b could be a target of mycolactone. The implication of the interaction can be experimentally evaluated to further understand its role in granule exocytosis impairment in Buruli ulcer.

## 1. Introduction

Buruli ulcer is a damaging skin disease caused by *Mycobacterium ulcerans* [1]. There are cases of Buruli ulcer reported worldwide, particularly in the West African region and some areas in Australia [2,3]. It is characterized by ulceration of subcutaneous fat, starting as small painless lesions which may eventually grow into large cutaneous ulcers and open wounds [4]. The ulcers and wounds if not properly treated usually leave irreversible and long-term disabilities on affected individuals [5]. 

*Mycobacterium ulcerans* is known to produce a lipid-like exotoxin called mycolactone [6]. Mycolactone disrupts important cellular functions in host cells and exhibits immunosuppressive properties even at low doses [6]. The toxin accumulates in the extracellular matrix and has the ability to passively permeate host cells due to its lipophilic properties [7]. Currently, the exact mechanism by which mycolactone contributes to the virulence of Buruli ulcer is not clear, however evidence of its action has been reported in different cell types such as the fibroblast and macrophage cell lines [8]. This implies that the toxin functions in multiple pathways and its cumulative effect results in Buruli ulcer virulence. 

As stated earlier, Buruli ulcer is characterized by lack of wound healing/repair which is accompanied by no sensation of pain in the wounded area [9,10]. From interactions with people affected by the ulcer, it was realized that almost all of them recall a trauma on the skin like a tiny cut which developed into a bigger wound. The ulcers are also characterized by the occurrence of a layer of dead cells found at the base of the wounds [11]. Wound healing is a complex process which normally occurs in four stages, namely hemostasis, inflammation, proliferation, and maturation [12]. Two major cells involved in the wound healing process are platelets and mast cells. 

As soon as there is injury, there is activation of platelets causing the secretion of bioactive substances such as serotonin (5-HT), ionized calcium, ATP and ADP stored in intracellular granules to facilitate wound healing. These molecules are necessary for hemostasis. Platelets also release mediators and proinflammatory factors which include cytokines and histamine for inflammation [13]. Mast cells are activated upon exposure to certain molecular signatures and release cytokines, histamines, and other molecular mediators necessary to begin inflammation [14,15]. A common characteristic of both cells is that they contain granules, within which the molecules to be released are stored. Upon activation, the contents of the granules are released through a mechanism called granule exocytosis [16,17].

The granular contents released by platelets and mast cells have been shown to play crucial roles in wound healing and pain perception. Recent work has shown that serotonin decreases apoptosis, increases cell survival, cellular proliferation, and accelerates cell migration significantly [18]. Most of the peripheral serotonin is stored in platelet dense granules, to balance the amount of free-flowing serotonin in the bloodstream [18]. Calcium released from the exocytosis of these platelets also plays an important role by serving as a modulator of keratinocyte proliferation and differentiation [19]. Calcium is also necessary for cell survival and the lack of it results in apoptosis [20]. 

The release of serotonin, histamine, and cytokines including other cellular changes stimulate the free nociceptor nerve endings which send impulses through the nerve cells for the perception of pain [21]. A nociceptor is a free nerve ending found in cutaneous muscle and visceral tissues [21]. Inflammatory pain is also characterized by an increase in cytosolic calcium ion concentration in dorsal root ganglion (DRG) neurons [22]. The increase in calcium levels is facilitated by transmitter release both in the periphery and at central terminals [22]. 

It has been shown that lack of platelet-mediated serotonin release causes impaired hemostasis, dysregulation of the remodeling phase of skin healing, and systemic immunosuppression [18]; this similarly happens during Buruli ulcer infections. Interestingly, it has also been reported that mycolactone does not affect the aggregation of platelets at the wound site of Buruli ulcer patients [3]. The then evidence points to the possibility of an interruption in the granule exocytosis mechanism, contributing to the lack of wound healing and pain in Buruli ulcer. 

Recent attempts to understand the toxin’s mechanism of action have shown mycolactone to bind to different receptors including neuron expressed angiotensin II receptors (AT2R), Wiskott-Aldrich Syndrome protein (WASP), and Sec61 translocon proteins [1,23,24]. Mycolactone induces analgesia by interacting with AT2R receptors in sensory neurons [1]. Recent work has also shown that it binds to WASP with an affinity 100-fold greater than its natural activator, leading to defective cell adhesion and eventually apoptosis [24]. For Sec61 translocon proteins, inhibition by mycolactone blocks co-translational translocation into the endoplasmic reticulum. A R66G mutation in Sec61 causes broad resistance to mycolactone [23,25]. None of these studies report a direct link to mycolactone interruption of the granule exocytosis mechanism. 

Granule exocytosis in both platelets and mast cells is facilitated by soluble n-ethylmaleimide-sensitive factor attachment protein receptors (SNAREs) [16,17]. SNARE proteins exist in two groups; v-SNAREs and t-SNAREs based on their locations; v-SNAREs are on the vesicle membrane while t-SNAREs are on the platelet membrane [17]. They form a coil structure, merging the vesicle membrane to the platelet cell membrane to release the granular contents [16,17]. In mast cells, there is also the merging of granules to form channels, contributing to the speed at which degranulation occurs [16]. There are proteins that regulate the binding of the t- and v-SNAREs in order to avoid the arbitrary release of granular content [17]. Important SNARE proteins and regulators that are present in platelets and mast cells include syntaxin, vesicle-associated membrane protein (VAMP) 8, synaptosomal-associated protein (SNAP) 23, syntaxin-binding protein 2 (Munc18b), Munc13-4, Rab27, and synaptotagmin-like protein (SLP). 

VAMPs form the largest group of v-SNAREs with VAMP8 being the most abundant and physiologically important in human platelets [17]. Loss of VAMP8 impairs granule exocytosis in mast cells and platelets [16,17]. Among the syntaxins, syntaxin 11 is essential for platelet granule exocytosis whilst syntaxin 3 and 4 are involved in degranulation of mast cells [16,17]. Loss of syntaxin 11 can cause familial hemophagocytic lymphohistiocytosis type 4 (FHL4) in humans. Syntaxins form complexes with SNAP23 and VAMP8 during fusion of the plasma and vesicle membranes. SNARE regulators in both mast cells and platelets comprise Munc18b, Munc13-4, Rab27, and synaptotagmin-like protein (SLP). Munc13-4 is an effector protein of Rab27 and SLPs as calcium sensors; they regulate the docking of the vesicle to the plasma membrane [16]. Loss of Munc13-4 and Rab27 causes defective granule exocytosis [17]. Munc18b, a syntaxin chaperone protein binds to syntaxin 11 in platelets and syntaxin 3 in mast cells [16,17]. Studies have reported that knockout of Munc18b leads to a decrease in intracellular syntaxin 11 and subsequently impairs dense granule exocytosis [17].

In this study, we investigated the binding of mycolactone to a SNARE protein/regulator and hypothesize its probable interruption of granule exocytosis. The studies involved docking mycolactone to selected SNARE proteins and regulators (VAMP8, syntaxin 11, SNAP23, Munc18b, and Munc13-4), as well as Sec61, WASP, and AT2R. Predicted binding affinities between mycolactone and the SNARE proteins and regulators were compared with those of Sec61, WASP, and AT2R. Molecular docking studies showed Munc18b to be a putative target of mycolactone, as such further studies on the binding interactions were undertaken. The structural stability of Munc18b-mycolactone complex was compared to that of Sec61, which is known experimentally to bind to mycolactone.

## 2. Results

### 2.1. Molecular Docking 

#### 2.1.1. Mycolactone has a Higher Binding Affinity for Munc18b than other SNARE Proteins

The binding affinity of mycolactone to important SNARE proteins and regulators crucial for granule exocytosis in both platelets and mast cells was investigated using molecular docking methods. Mycolactone was docked blindly against VAMP8, SNAP23, syntaxin 11, Munc13-1 (Munc13-4 isoform), and Munc18b. Molecular docking studies were done using AutoDock Vina [26] in PyRx version 0.8 [27] with exhaustiveness of 20 for all the respective docking procedures. Binding energies of −4.4 kcal/mol, −5.7 kcal/mol, −6.0 kcal/mol, −6.2 kcal/mol, and −8.5 kcal/mol were obtained for SNAP23, VAMP8, syntaxin 11, Munc13-1, and Munc18b, respectively. Normally, a compound is predicted to have activity against a protein when it has binding energy less than −6.0 kcal/mol [28], thus the results show mycolactone to have the strongest binding affinity to Munc18b. Moreover, characterization of binding interactions show that mycolactone forms more intermolecular interactions composed of hydrogen and hydrophobic bonds with Munc18b than any of the other SNARE proteins (Table 1).

#### 2.1.2. Predicted Binding Energies for Known Mycolactone Receptors (Sec61 and AT2R) and Munc18b Are High

The protein structures of known mycolactone targets comprising Sec61, WASP, and AT2R were also retrieved from the protein data bank and mycolactone was docked against them, respectively. The docking parameters used for the targets were similar to the previous studies, except that this time the grid box was adjusted to cover the active site pockets/domains of the respective targets. The binding energies obtained were −9.0 kcal/mol, −8.9 kcal/mol, and −7.1 kcal/mol for AT2R, Sec61, and WASP, respectively (Table 2), which similarly exceeds the threshold of −6.0 kcal/mol.

### 2.2. Molecular Dynamics Simulation

#### 2.2.1. RMSD and RMSF Graphs show Instability in Munc18b’s Structure after the Binding of Mycolactone

**Figure 1:**
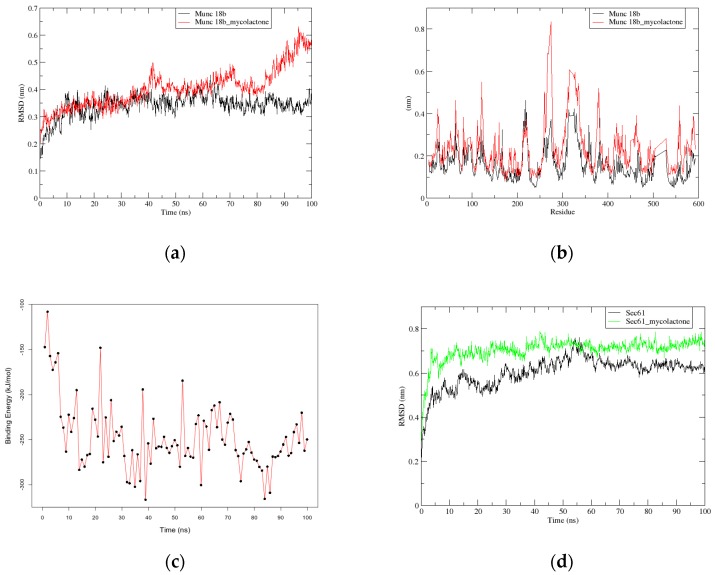
Graphs of root mean square deviation (RMSD), root mean square fluctuation (RMSF), and binding energy of Munc18b and its complex: (**a**) RMSD versus time graph of the backbone atoms of Munc18b-mycolactone complex (red) relative to Munc18b (black) over 100 ns; (**b**) RMSF graph plot of residues of Munc18b-mycolactone complex (red) relative to residues of Munc18b (black) alone; (**c**) binding energy versus time graph of Munc18b-mycolactone complex over 100 ns simulation; and (**d**) RMSD versus time graph of the backbone atoms of Sec61-mycolactone complex (green) relative to Sec61 (black) over 100 ns.

Structural studies of Munc18b complexed with mycolactone and another complex of mycolactone with its known receptor, Sec61, were further investigated using molecular dynamics (MD) simulations. Simulations of 100 ns were separately carried out for the Munc18b and Sec61 native proteins, and the Munc18b-mycolactone and Sec61-mycolactone complexes. The root mean square deviation (RMSD) values were calculated for the positional differences of the backbone atoms over time for the native proteins and the complexes. A stable RMSD was obtained for the Munc18b native protein compared to that of the Munc18b-mycolactone complex. Their RMSDs fluctuated between 0.3 and 0.4 nm in the initial 30 ns of the simulation (Figure 1a). However, the RMSD of the Munc18b-mycolactone complex experienced slight fluctuations after 30 ns until about 85 ns, where it began to deviate widely. This occurrence is quite the opposite for Sec61, since the RMSD of the native Sec61 protein structure experienced more fluctuations than that of the Sec61-mycolactone complex. Sec61-mycolactone complex was observed to fluctuate around 0.7 nm from the first 10 ns of the simulation, whilst the RMSD of Sec61 protein alone steadily rose from 0.5 nm to 0.7 nm till the 55^th^ ns, where it drops slightly till it stabilizes at 0.6 nm in the final 20 ns of the simulation (Figure 1d). This shows that mycolactone forms a very stable complex with Sec61. 

**Figure 2:**
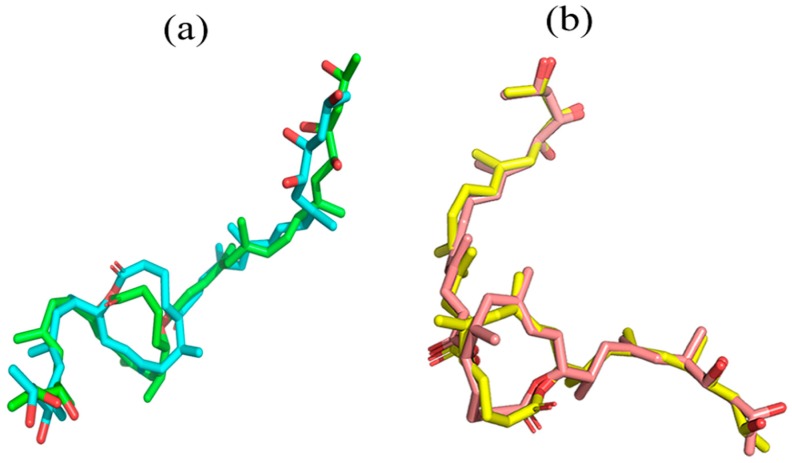
Superimposed structural poses of mycolactone within Munc18b and Sec61 complexes. (**a**) Superimposition of mycolactone pose before (green) and after (cyan) molecular dynamics (MD) simulation for Munc18b complex; and (**b**) superimposition of mycolactone pose before (pink) and after (yellow) MD simulation for Sec61 complex. RMSD values of 1.859 and 0.923 Å were obtained for Munc18b (**a**) and Sec61 (**b**) complexes, respectively.

Original poses of mycolactone within Munc18b and Sec61 complexes were compared to the final poses obtained after MD via superimposition using LigAlign [32] (Figure 2). This was to investigate if the ligand experienced any change in conformation which may support the observed deviations. An RMSD value of 1.859 Å was obtained between the pre-MD and post-MD Munc18b-mycolactone complexes. Also, an RMSD value of 0.923 Å was obtained between pre-MD and post-MD Sec61-mycolactone complexes. Since the respective RMSDs fell below the similarity threshold of 2 Å [33], it indicates that no appreciable conformational changes were experienced by the ligand but possibly a change in the structural integrity of the protein. To further investigate the wide deviation in the RMSD of the Munc18b-mycolactone complex, fluctuations in the movement of residues before and after the binding of mycolactone to the protein were analyzed by calculating the root mean square fluctuation (RMSF) of the residues (Figure 1b). High random mobility of residues in the complex was observed compared to that of the protein alone as RMSF plots showed larger values. Residues within the range of 100–400 were observed to generally show high fluctuations. Residues in the range of 250–350 showed the highest peaks with values above 0.6 nm (Figure 1b). This shows that the binding of mycolactone to the protein increases the flexibility of the residues which further affirms changes in the protein structure as observed from the RMSD graphs. 

#### 2.2.2. G_mmpbsa Calculations show Strong Binding between Mycolactone and Munc18b 

The g_mmpbsa [34,35] which uses the MM-PBSA method was used to calculate the contribution of van der Waal energy, electrostatic energy, polar solvation energy and non-polar solvation energy to the overall binding energy. Molecular mechanics energies coupled with the Poisson–Boltzmann or generalized Born and surface area continuum solvation (MM-PBSA and MM-GBSA) are known methods used to estimate free binding energies of the binding of small molecular compounds to macromolecules [36]. The energy terms were calculated for the whole 100 ns production run as 100 snapshots were taken at an interval time of 1ns with default settings. The binding energy results obtained were assessed in terms of average energy and standard deviation. A binding energy graph over the whole simulation time was plotted. It was observed that the binding energy of the complex within the first 10ns of the simulation dropped sharply from −100 kJ/mol to around −260 kJ/mol, after which it averaged within the range of −200 to −300 kJ/mol (Figure 1c). An average binding energy of −247.571 ± 37.471 kJ/mol was obtained at the end of the simulation which shows strong binding between mycolactone and Munc18b. The binding energy is largely contributed by van der Waal energies and slightly by nonpolar solvation energies and electrostatic energies. Polar solvation energies, on the other hand, were unfavorable for the binding energy (Table 3).

### 2.3. Critical Residues for Mycolactone and Munc18b Binding

**Figure 3:**
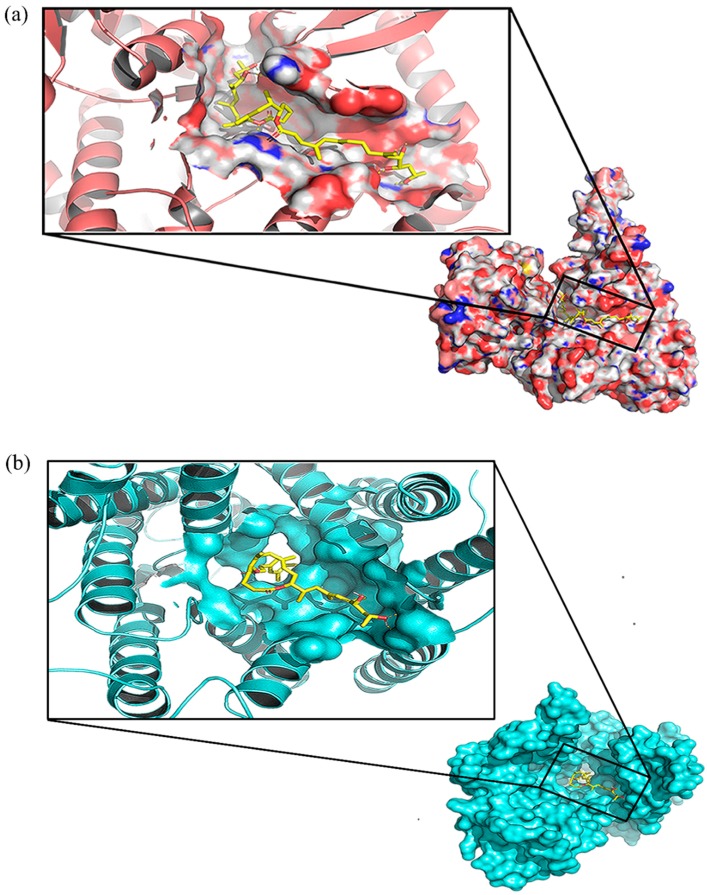
The binding pose of mycolactone in the central cavity of Munc18b (**a**) and Sec61 (**b**). Mycolactone is shown in yellow sticks. Images were generated with PyMOL [38].

**Figure 4:**
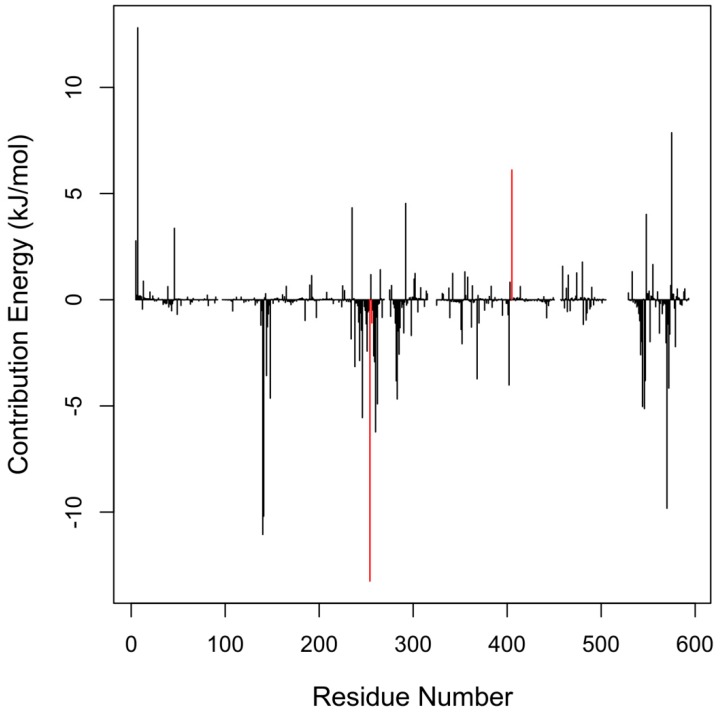
Molecular mechanics Poisson-Boltzmann surface area (MM-PBSA) plot of binding free energy contribution per residue of Munc18b-mycolactone complex. Fluctuations by predicted critical residues are highlighted in red.

From visualization of the Munc18b-mycolactone complex, mycolactone is observed to be binding to the central cavity of the protein (Figure 3a). The central cavity has been reported to be the largest domain, sitting between two structural domains that are conserved and crucial for Munc18 protein function [37]. Binding interaction studies were performed on the Munc18b-mycolactone complex retrieved after molecular docking and also after undergoing molecular dynamics since protein flexibility is introduced during molecular dynamics simulations. This was done to determine important residues necessary for the binding of mycolactone. The interacting residues observed after molecular docking and molecular dynamics are listed in Table 4. Tyr254, Leu256, and Arg405 were observed to interact with mycolactone in both states of the complex, however, Arg405 and Leu 256 are observed to be forming hydrophobic bonds in the post-MD complex and hydrogen bonds in the pre-MD complex (Table 4). Energy contribution of all residues was investigated by calculating the MM-PBSA decomposition of the binding energy (Figure 4). A majority of residues contributed minimal energies, but some residues contributed high negative and positive energies. Tyr254 contributed the highest negative energy of value −13.2513 kJ/mol. The other two predicted critical residues comprising Leu256 and Arg405 also contributed −1.0928 and 6.1118 kJ/mol, respectively. Worth mentioning are residues that contributed energies <−5.0 and >5.0, but nonetheless these were not part of predicted critical residues. These residues include Lys7, Tyr140, Glu141, Glu260, Glu246, Met544, Glu546, Arg575, and Ile570 (Table 5). 

## 3. Discussion

Mycolactone has been shown to passively enter cells and interact with membranes [7]. The toxin is shown to prefer organic environments to aqueous and has the tendency to behave as a linactant [7]. This suggests that interference in the fusion of vesicle and plasma membranes by mycolactone is plausible. The platelet granule exocytosis pathway involves granule docking, priming, and finally membrane fusion as well as cargo release [17]. Proteins located on the vesicle membrane, which are Rab27b, SLP, and Munc13-4 are required for granule docking. Activation of the platelets leads to change in conformation in the syntaxin protein which is sequestered by Munc18b [17]. This results in priming, where there is the formation of four helical bundles from the binding of syntaxin 11, SNAP23, and VAMP8. This fusion ultimately leads to the formation of the membrane fusion pore and cargo release [17]. 

Degranulation/granule exocytosis of mast cells involve a similar mechanism as described for platelets. However, mast cell secretory granules are also able to merge together to create channels [16]. This normally happens after the vesicle fuses with the plasma membrane in sequential exocytosis or before the vesicle fuses with the plasma membrane in multivesicular exocytosis [16]. The additional mechanism allows mast cells to release high amounts of granular contents upon activation. Syntaxin 3(STX3) and Munc18b are necessary for both granule-granule and granule-plasma membrane fusion, as such they are present on both granule membranes and the plasma membrane in mast cells [16]. 

From the docking results, it was observed that mycolactone has a higher affinity towards Munc18b than the rest of the SNARE proteins. Also, the binding energy obtained for mycolactone against Munc18b exceeded the threshold for a compound to be considered active against a protein (<−6.0 kcal/mol), whilst those of VAMP8 and SNAP23 were below the threshold. Docking against syntaxin 11 (STX11) and Munc13-1 (Munc13-4 isoform), on the other hand, produced binding energies of −6.0 kcal/mol and −6.2 kcal/mol respectively, although close to the threshold, makes Munc18b a stronger candidate to be considered a target of mycolactone. As earlier mentioned, AT2R, Sec61, and WASP are reported targets of mycolactone [1,23,24]. This was corroborated by the binding energies obtained for them after docking, which fell well below −6.0 kcal/mol (Table 2). The docking pose of Sec61-mycolactone complex is shown in Figure 3b. Also, there were no wide differences in the binding energies obtained for Sec61, AT2R, and Munc18b, indicating possible roles in the binding mechanism of mycolactone. Based on these observations, we focused on the feasibility of Munc18b to be a target of mycolactone.

For a ligand to effectively interact with its receptor, it is expected that it docks to an active pocket and interacts with critical residues necessary for protein-ligand interactions. This requirement is met in Munc18b and the mycolactone binding interaction. Upon visualization and analysis of energy decomposition as well as binding interactions of the Munc18b–mycolactone complex, it was shown that mycolactone docks within the central cavity and shares binding interactions with Tyr254, Leu256, and Arg405 (Figure 3 and Figure 4, Table 4). The central cavity forms part of two important structural areas reported to be crucial for Munc18 protein functions, the subsequent area is an N-peptide binding site [37]. Also, recent work has shown that mutations in Arg405 lead to a complete loss of interaction between Munc18b and syntaxin 11 [37,39]. This indicates that binding of mycolactone to Munc18b may interfere with the protein’s normal function.

Molecular dynamics simulations performed on the complex further supported our suspicions. Changes in the structural integrity of the protein due to the binding of mycolactone can be observed from the RMSD and RMSF graphs (Figure 1a,b), as there are major fluctuations in the protein’s backbone and residue positions for the Munc18b-mycolactone complex’s system as compared to that of the protein alone. Interestingly, g_mmpbsa energy calculations over the entire simulation period of the complex show lower binding energy towards the end of the simulation, indicating strong binding between the mycolactone and Munc18b largely contributed by Van der Waal forces (Table 3).

To the best of our knowledge, no compound appears to have been reported to inhibit interactions between Munc18b and syntaxin. However, speculations have been that the binding of an effector molecule to Munc18a (a highly conserved isoform of Munc18b) could cause a rotation in its domain and thus the release of syntaxin [40]. Such an occurrence in Munc18b can inhibit both granule-granule and granule-plasma membrane fusion processes which will impair the release of granular contents. Mutations in Munc18b also leads to conformational change, preventing the binding of syntaxin, which also results in impaired platelet exocytosis [37,39]. From the observations made, it is reasonable to conclude that Munc18b is a plausible target for mycolactone. 

Based on these findings, we hypothesize that mycolactone impairs the release of granular contents that facilitate wound healing and pain perception by inhibiting the syntaxin chaperone protein, Munc18b. For Sec61, by its action in the translocation of proteins into the endoplasmic reticulum, at what point it has an effect in the wound healing processes cannot be ascertained. However, it could be upstream by blocking the synthesis and release of platelets activation factor or further downstream via the plethora of mechanisms of cells activations and proteins release. It is important to mention that the findings of the study cannot conclusively confirm the validity of the hypothesis since the work is computational and predictive. It is suggested that experimental studies be conducted to validate the claims made.

## 4. Conclusions

This study investigated the possible binding of mycolactone to SNARE proteins/regulators and hypothesized its possible implications in granule exocytosis. Molecular docking studies predicted a high binding affinity with binding energy of −8.5 kcal/mol for mycolactone against Munc18b, implying it is a plausible target of mycolactone. The binding affinity was appreciably higher than those predicted for the other SNARE proteins and regulators comprising VAMP8 (−5.7 kcal/mol), syntaxin 11(−6.0 kcal/mol), the isoform of Munc13-4–Munc13-1(−6.2 kcal/mol), and SNAP23 (−4.4 kcal/mol). MM-PBSA binding energy calculations performed with molecular dynamics simulations also revealed strong binding between mycolactone and Munc18b with an average binding energy of −247.571 ± 37.471 kJ/mol. Also, structural studies of Munc18b with and without a bound mycolactone showed changes in the protein’s conformation due to the binding of mycolactone. Critical residues for the binding of mycolactone were also predicted; these residues include Tyr254, Leu256, and Arg405. Arg405 is reported as an important residue whose mutation could result in impaired granule exocytosis. The predictions made assert the hypothesis that mycolactone interferes in the granule exocytosis processes by binding to Munc18b, causing impairment of wound healing processes. Further experimental studies are encouraged to understand mycolactone’s role in granule exocytosis.

## 5. Materials and Methods 

### 5.1. The Crystal Structures

The 3D crystal structural coordinates file of human syntaxin-binding protein 2 (Munc18b) was retrieved from Protein Data Bank, PDB (www.rcsb.org, PDB ID: 4CCA) [41]. It was solved at a resolution of 2.60 Å using x-ray diffraction. Some residues of Munc18b were not located in the x-ray diffraction experiment and, thus, were added and remodeled using the Swiss-PdbViewer [42]. The structural coordinate file of mycolactone B was retrieved from the PubChem database with CID: 5282079. Mycolactone B was chosen because it is one of the best studied of the mycolactone isomers [7]. In addition to the Munc18b protein, the 3D crystal structural coordinates files of two other SNARE proteins, namely synaptosomal-associated protein 23 (SNAP-23) and vesicle-associated membrane protein 8 (VAMP8) were also retrieved from PDB with PDB IDs 1NHL and 4WY4, respectively [43,44]. The 3D crystal structure of Munc13-1 of *Rattus norvegicus* (PDB ID: 2CJT) [45], which is an isoform of Munc13-4 was also retrieved from PDB and in this case too, some residues were not located in the file, thus, they were added and remodeled as described above. Another SNARE protein, syntaxin-11, whose structure was not solved experimentally, was modeled using syntaxin1 (PDB ID: 4JEH) [46] as a homolog on the SWISS-MODEL server [47]. This homolog had a sequence identity of 38% and a query coverage of 62% to the syntaxin-11 protein. Also, the crystal structures of three proteins comprising Wiskott-Aldrich Syndrome protein (WASP, PDB ID: 1EJ5), Protein transport protein SEC61 (PDB ID: 3JC2), and angiotensin II type 2 receptor (AT2R, PDB ID: 5UNG), which have been reported to have interactions with mycolactone were retrieved from PDB [7,23,24,48,49,50].

### 5.2. Molecular Docking

All the molecular structures were prepared via PyRx [27] for molecular docking using AutoDock Vina [26]. In the preparation, Munc18b and other protein structural files were converted to PDBQT format as accepted by the AutoDock Vina and the molecular structure of mycolactone was minimized with the universal force field and further converted to PDBQT format. Ligand restriction grid boxes were set for Sec61 ((center = (398.36, 287.68, 341.27), size = (44.66, 38.81, 41.59)); WASP ((center = (−6.11, −0.46, 0.88), size = (63.64, 48.37, 34.14)); and AT2R ((center = (11.37, 5.46, −17.76), size = (28.82, 34.06, 25.00)), covering their active sites. Also, the ligand restriction grid boxes for the other proteins covered the entire protein structures. The exhaustiveness was set to twenty in each case, this allows for a global search of the mycolactone compound to dock to a more comfortable place on the various proteins. After the molecular docking, Ligplot+ [51] was used to characterize the binding interactions between the human Munc18b and mycolactone. Furthermore, the binding energies of mycolactone with the other proteins were also evaluated after the docking protocol.

### 5.3. Molecular Dynamics Simulations

All molecular dynamics (MD) simulations were performed for 100 nanoseconds with the GROMOS96 43a1 force field and SPC water model using GROMACS version 2018 [52]. MDs were ran on a Dell EMC high performance computing (HPC) system composed of CentOS 7 operating systems, 6 nodes, 12 GPUs, 216 CPUs and storage of 277 TB, located at the West African Centre for Cell Biology of Infectious Pathogens (WACCBIP), University of Ghana, Accra. An initial MD was done using the downloaded Munc18b coordinate file from PDB as the starting point, and another MD was done using the docked complex of Munc18b and mycolactone. The simulations were done in a dodecahedron box of size 1.0 nm, solvated with SPC water and neutralized by adding 10 sodium ions. For the docked complex MD, the topology file for mycolactone was generated using PRODRG2 server with the settings herein (Chirality: Yes, Charges: Full, EM: No). The PDB structure of mycolactone was used as input to the server and topologies were generated for the ffgmx GROMACS (GROMOS96) force field. Energy minimization was done at 1000 steps using the steepest descent algorithm. Position restrains were applied to the Munc18b and the mycolactone, after which a temperature equilibration at 300 K followed by a pressure equilibration at 1 bar was performed for 50,000 ps each. The production MD runs were then performed for 100 ns, keeping the temperature at 300 K and the pressure at 1 bar. Xmgrace [53] was used to plot the graphs generated from the MD simulations. MD simulations for Sec61 and Sec61-mycolactone complex followed a similar procedure, neutralization was however achieved by adding five chlorine ions. The pre-MD pose of mycolactone was superimposed over that of post-MD within Munc18b using LigAlign [32]. 

### 5.4. Binding Energy Calculations using the MM-PBSA Method

The binding energies of the Munc18b-mycolactone complex were calculated with g_mmpbsa [34,35] over a 100 ns time stamp in steps of 1 ns for the MD simulation. R programming package [54] was used to plot the graphs from the MM-PBSA calculations. The details of the molecular dynamics parameters and index data are shown in Supplementary Files 1 and 2, respectively.

## Figures and Tables

**Table 1:** A table showing residues involved in H-bond and hydrophobic interactions between mycolactone and soluble n-ethylmaleimide-sensitive factor attachment protein receptor (SNARE) proteins. More residues in Munc18b are involved in H-bond interactions with mycolactone than any of the other SNARE proteins.

SNARE Protein Target	H-Bond Residues	Hydrophobic Bond Residues
Munc18b	His245, Asp255, Leu256, Asp262, Arg402, Arg405, Thr574, Arg575	Lys7, Ser43, Leu138, Tyr140, Ser146, Tyr254, Gln261, Val361, Ile570, Thr572, Asp578.
SNAP23	Ser39	Gln40, Gly43, Thr46, Ile47, Leu50, Asp51, Lys54
VAMP8	Thr48, Ser55	Leu51, Glu52, Thr54, Glu56, Phe58, Lys59
Syntaxin 11	None	Gln140, His144, Asn147, Met151, Arg154, Glu218, Ile221, Arg222, Phe228, Leu229, Ala232, His255
Munc13-4	Arg86, Glu95, Asp124, Ala125	Val78, Trp79, Ile80, Thr84, Ile85, Leu97, Thr98, Leu99, Asp100, Phe118

**Table 2:** A table showing all the proteins that were docked with mycolactone. The binding energies obtained are in kcal/mol alongside their locations in the human cells and their respective physiologic functional roles.

Protein	Binding Energy /kcal/mol	Location in Human Cells	Functional Roles
Munc18b	−8.5	Localized on the plasma membrane in platelet cells and vesicle membranes of mast cells	Interacts with STX 3 in mast cells and STX11 in platelets to regulate granule exocytosis [16,17].
Sec61	−8.9	Endoplasmic reticulum(ER)	Responsible for protein translocation into the endoplasmic reticulum [29]
Wiskott-Aldrich protein (WASP/NWASP)	−7.1	Cytoskeleton	Dynamic extensive alteration of actin filament through its interaction with Arp2/3 complex [30]
Type 2 angiotensin II receptor (AT2R)	−9.0	Plasma membrane Sensory neurons	Involved in cell proliferation and functional inhibition of ERK2 receptor [31]; involved in nociception and neuronal regeneration [1].
Vesicle associated membrane protein 8 (VAMP8)	−5.7	Vesicular, Secretory granules	Forms an extended parallel four alpha-helical trans-SNARE complex with STX11 and SNAP23 upon stimulation, causing membrane fusion and driving platelet exocytosis [17].
Syntaxin 11 (STX11)	−6.0	Plasma membrane	Interacts and binds selectively with SNAP23 and VAMP8 to form a complex during membrane fusion and they facilitate granule exocytosis [17].
N-ethylmaleimide-sensitive factor attachment protein 23 (SNAP23)	−4.4	Plasma membrane	Highly involved in membrane fusion regulation during granule exocytosis in platelet and mast cells. Usually binds to VAMP8 and STX4 in mast cells and VAMP8 and STX 11 in platelets during exocytosis [16,17].
Munc13-4 (Isoform Munc13-1 was used for docking)	−6.2	Highly localized on the plasma membrane, endosome, lysosome, and the cytoplasm	Involved in granule maturation, docking, and vesicle fusion: playing a major role in vesicle priming. They bind to STX4 in mast cells during degranulation process [16].

**Table 3:** A table showing binding energy and its contributing energy terms of Munc18b-mycolactone complex. The values are presented in average ± standard deviations in kJ/mol.

Energy Terms	Munc18b-Bound Energy Values (kJ/mol)
van der Waal energy	−313.404 ± 35.505
Electrostatic energy	−49.944 ± 28.281
Polar solvation energy	144.781 ± 43.370
Nonpolar solvation energy	−29.005 ± 3.066
Binding energy	−247.571 ± 37.471

**Table 4:** A table showing hydrogen and hydrophobic bonds formed between mycolactone and Munc18b before and after MD simulations. Tyr254, Leu256, and Arg405 are common residues in pre-MD and post-MD complexes.

**Molecular Interactions before MD Simulations**		
Protein Complex	H-Bond Residues	Hydrophobic Bonded Residues
Munc18b-Mycolactone complex	His245, Asp255, Leu256, Asp262, Arg402, Arg405, Thr574, and Arg575.	Lys7, Ser43, Leu138, Tyr140, Ser146, Tyr254, Gln261, Val361, Ile570, Thr572, and Asp578.
**Molecular Interactions after MD Simulations**		
Protein Complex	H-Bond Residues	Hydrophobic Bonded Residues
Munc18b-Mycolactone complex	Val542, Leu571	Tyr140, Val144, Pro242, Leu243,Leu244,His245,Ala251, Tyr254,Asp255,Leu256, Tyr401,Arg405, Gly541, Ser567, Ile570, Thr572, Pro573

**Table 5:** A table showing residues contributing large amount of negative (<−5.0) and positive (>5.0) energies towards mycolactone binding relative to the total binding energy. Among them are predicted critical residues Tyr254 and Arg405.

Residues	Energies <−5.0 kJ/mol	Residues	Energies >5.0 kJ/mol
Met544	−5.0279	**Arg405**	6.1118
Glu546	−5.1263	Arg575	7.8640
Glu246	−5.5520	Lys7	12.8054
Glu260	−6.2233		
Ile570	−9.8204		
Glu141	−10.1902		
Tyr140	−11.0537		
**Tyr254**	−13.2513		

## Supplementary Materials

The following are available online at https://www.mdpi.com/2072-6651/11/3/181/s1, File S1: Molecular dynamics parameters in .mdp format. File S2: The index file in .ndx format.
